# Supplementary material for: Restoration of the healing microenvironment in diabetic wounds with matrix-binding IL-1 receptor antagonist
Source: Commun Biol. 2021 Mar 26;4:422. doi: 10.1038/s42003-021-01913-9 (PMC7998035; doi:10.1038/s42003-021-01913-9)
Supplement: Supplementary file 2 — Supplementary Information [file 42003_2021_1913_MOESM2_ESM.pdf]

Supplementary information

Restoration of the healing microenvironment in diabetic wounds with matrix-binding IL-1 receptor antagonist

Jean L. Tan[1,#], Blake Lash[1,2,#], Rezvan Karami[1], Bhavana Nayer[1], Yen-Zhen Lu[1], Celeste Piotto[1], Ziad Julier[1], Mikaël M. Martino[1*]

[1]European Molecular Biology Laboratory Australia, Australian Regenerative Medicine Institute, Monash University, Melbourne, VIC, Australia

[2]Department of Biological Engineering, Massachusetts Institute of Technology, Cambridge, MA, USA

[#] Equal contribution

*Corresponding author: mikael.martino@monash.edu

This PDF file includes:

- Supplementary Figures 1–10.
- Supplementary Table 1.

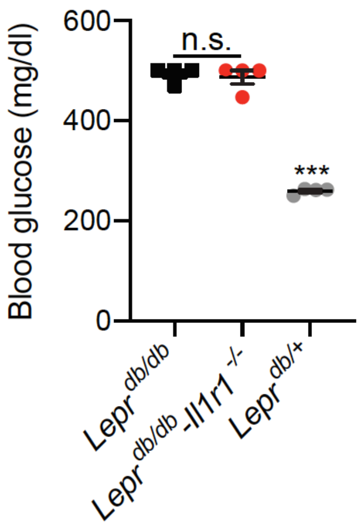

Supplementary Figure 1. Blood glucose levels of *Lepr*$^{db/db}$ and *Lepr*$^{db/db}$*-Il1r1*$^{-/-}$ mice. Blood glucose of 14-week old mice was measured. The diabetic phenotype (high blood glucose) was not altered by IL-1R1 deficiency. *n* = 4 mice per group. Data are means ± SEM. One-way ANOVA with Bonferroni *post hoc* test for pair-wise comparisons. ****P* ≤ 0.001, n.s. = non-significant.

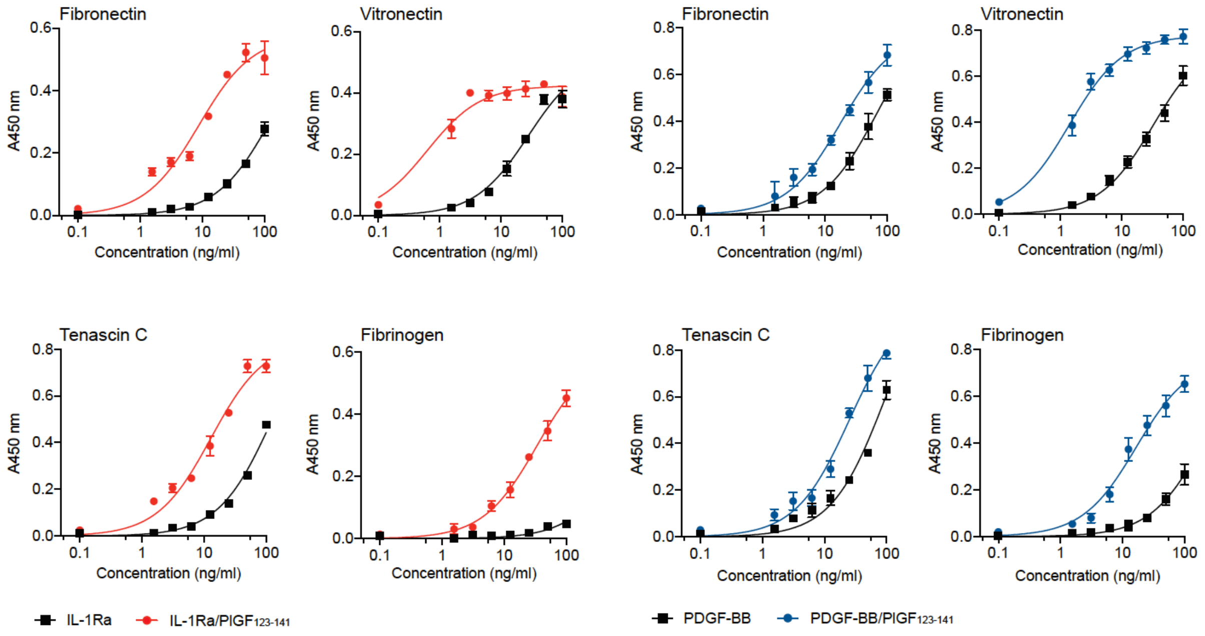

Supplementary Figure 2. Binding of PlGF$_{123-141}$-fused IL-1Ra and PDGF-BB to ECM proteins. ELISA plate wells were coated with ECM proteins and further incubated with PlGF$_{123-141}$-fused or wild-type proteins. Graphs show signals given by an antibody detecting IL-1Ra or PDGF-BB. The signals were fitted by non-linear regression to obtain the dissociation constant (K_d) using A450 nm = B_{max}*[protein]/(K_d + [protein]) where [protein] is the concentration of IL-1Ra, IL-1Ra/PlGF$_{123-141}$, PDGF-BB, or PDGF-BB/PlGF$_{123-141}$. Representative binding curves are shown.

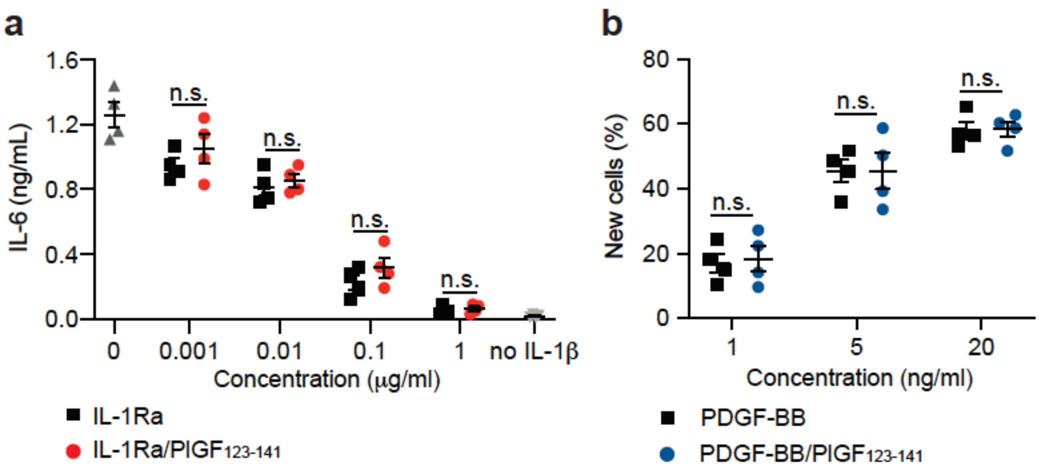

Supplementary Figure 3. Fusing PlGF$_{123\text{-}141}$ to IL-1Ra and PDGF-BB does not impair activity. (**a**) Bone marrow-derived macrophages were co-stimulated with IL-1β (1 ng/ml) and IL-1Ra variants at increasing concentrations (0 to 1 μg/ml). The negative control was no IL-1β treatment. The ability of IL-1Ra variants to inhibit IL-1β was assessed by measuring the release of IL-6 by macrophages 24 h after stimulation. n = 4. (**b**) Dermal fibroblasts were cultured in basal media (2% serum) and stimulated with PDGF-BB variants at increasing concentrations. The percentage increase in cell number was measured after 3 days. n = 4. For both panels data are means ± SEM. Two-tailed Student's *t* test. n.s. = non-significant.

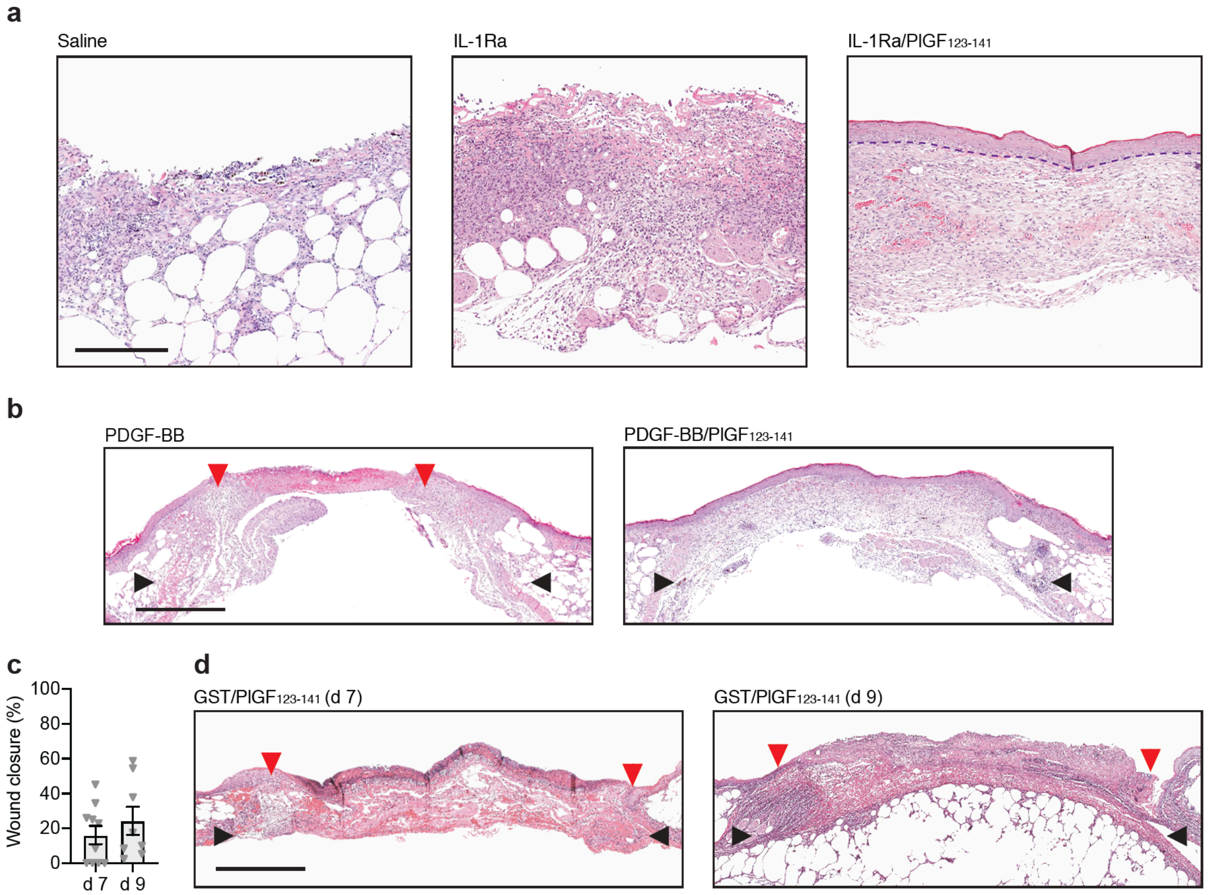

Supplementary Figure 4. Wound healing induced by IL-1Ra and PDGF-BB variants. (**a-d**) Full-thickness wounds in *Lepr*$^{db/db}$ were treated with IL-1Ra variants, PDGF-BB variants, or GST/PlGF$_{123\text{-}141}$ control protein (0.5 μg of wild-type, equimolar of engineered version or GST control). Representative histology (hematoxylin and eosin staining) 7 or 9 d post-treatment (a, b, and d). Black arrows indicate wound edges and red arrows indicate tips of epithelium tongue. The epithelium (if any) appears in purple as a homogeneous keratinocyte layer on top of the wounds. The delimitation between the dermis and the epithelium is shown as a dashed purple line for the IL-1Ra/PlGF$_{123\text{-}141}$ condition. The granulation tissue under the epithelium contains granulocytes with dark-purple nuclei. Fat tissue appears as transparent bubbles. Scale bar = 0.5 mm in (a), 1 mm in (b) and (d). Wound closure following treatment with GST/PlGF$_{123\text{-}141}$ evaluated by histomorphometric analysis of tissue sections in (c). Data are means ± SEM, *n* = 10 wounds for d 7 and 8 wounds for d 9. The extent of wound closure induced by GST/PlGF$_{123\text{-}141}$ is similar to the one induced by saline control (shown in Fig. 3a,b), demonstrating that PlGF$_{123\text{-}141}$ has no influence on wound closure.

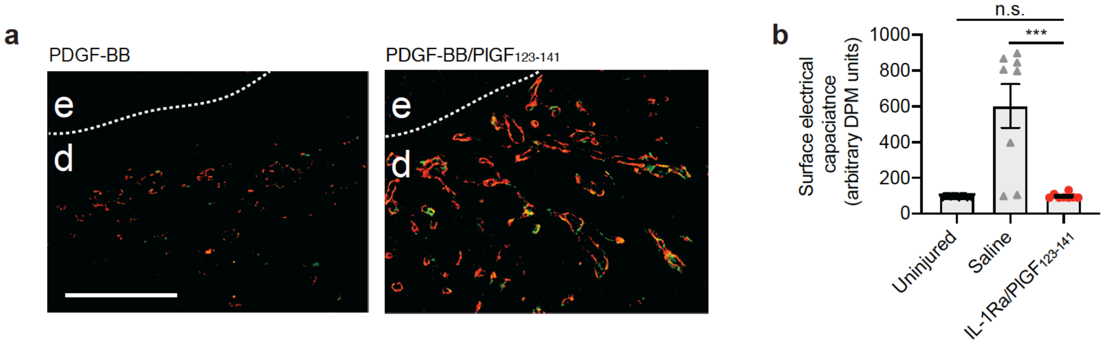

Supplementary Figure 5. Angiogenesis and epithelial barrier integrity in wounds treated with IL-1Ra and PDGF-BB variants. (**a** and **b**) Full-thickness wounds in *Lepr*$^{db/db}$ were treated with PDGF-BB or IL-1Ra variants (0.5 µg of wild-type, equimolar of engineered version). Representative images of angiogenesis at d 9 post-treatment assessed by immunostaining of wound sections for CD31 (endothelial cells, red) and desmin (smooth muscle cells, green) in (a). Dashed lines indicate separation between epidermis "e" and dermis "d". Scale bar = 0.2 mm. Measurement of surface electrical capacitance was used to evaluate the integrity of the skin barrier 9 d post-treatment with saline and IL-1Ra/PlGF$_{123-141}$. The graph in (b) shows the surface electrical capacitance given in dermal phase meter arbitrary units. Data are means ± SEM. $n = 8$. One-way ANOVA with Bonferroni *post hoc* test for pair-wise comparisons. ***$P \leq 0.001$, n.s. = non-significant.

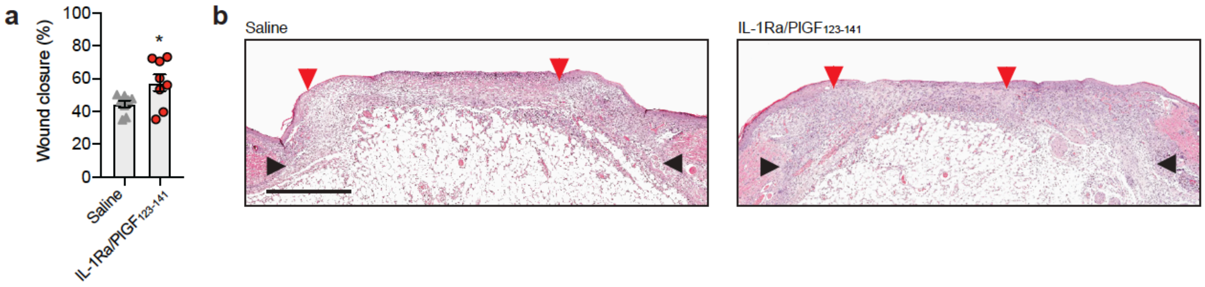

Supplementary Figure 6. Wound healing induced by super-affinity IL-1Ra in non-diabetic mice. (**a** and **b**) Splinted full-thickness wounds in wild-type mice (C57BL/6) were treated with saline or IL-1Ra/PlGF$_{123\text{-}141}$ (0.61 μg). Wound closure 6 d post-treatment evaluated by histomorphometric analysis of tissue sections in (a). Data are means ± SEM. n = 8 wounds per condition. Two-tailed Welch's t test. *$P \leq 0.05$. Representative histology (haematoxylin and eosin staining) 6 d post-treatment shown in (b). Black arrows indicate wound edges and red arrows indicate tips of epithelium tongue (stained in purple). The granulation tissue under the epithelium contains granulocytes with dark-purple nuclei. Fat tissue appears as transparent bubbles. Scale bar = 1 mm.

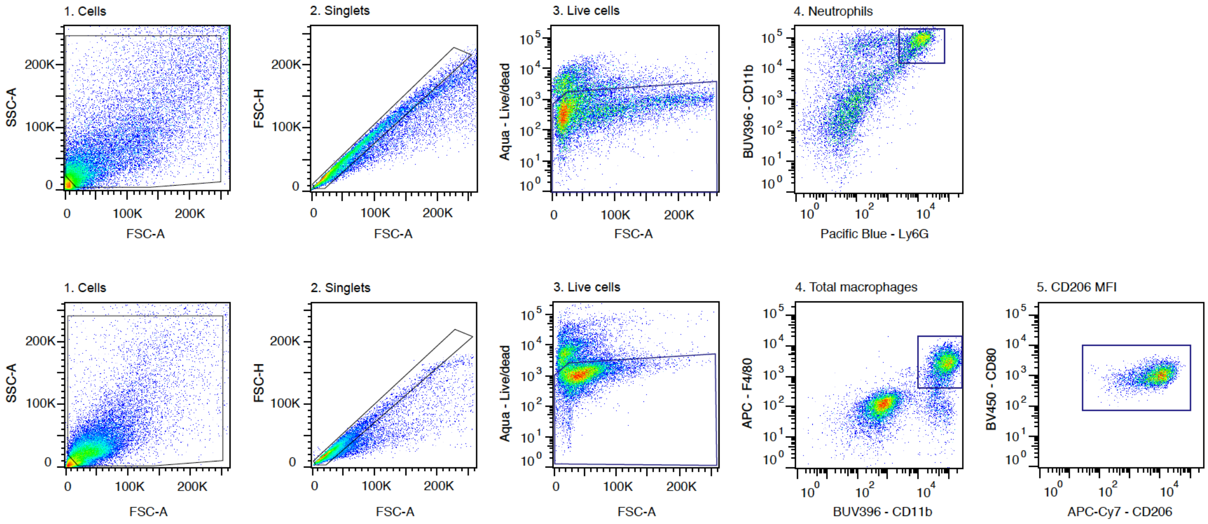

Supplementary Figure 7. Gating strategy to analyse wound neutrophils and macrophages. Step by step representative flow cytometry dot plots are shown for the neutrophil and macrophage panels. MFI, median fluorescence intensity.

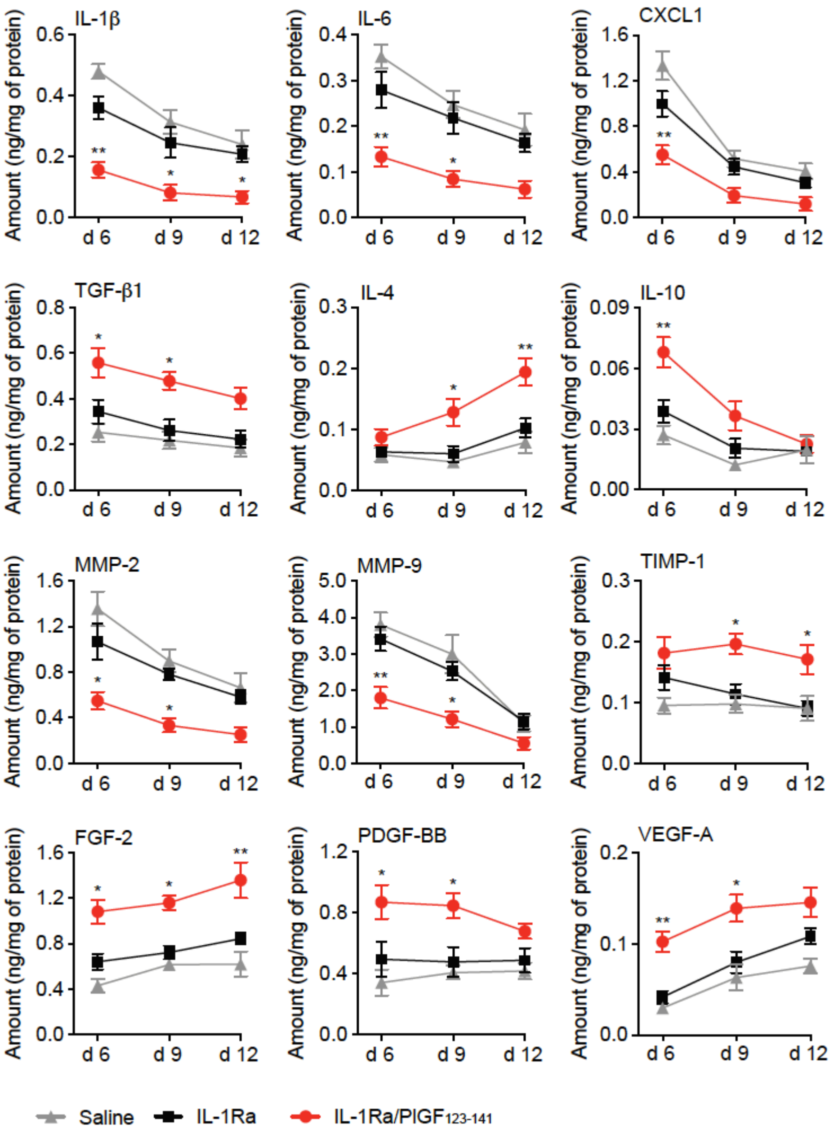

Supplementary Figure 8. Treatment with super-affinity IL-1Ra leads to a pro-healing biochemical microenvironment. Full-thickness wounds were created in *Lepr*$^{db/db}$ mice and treated with saline control or IL-1Ra variants (0.5 μg of wild-type, equimolar IL-1Ra/PlGF$_{123-141}$). Wound tissues were collected at day 6, 9, and 12 post-treatment and concentrations of cytokines, matrix metalloproteinases (MMPs), metallopeptidase inhibitor 1 (TIMP-1), and growth factors were measured by ELISA. The graphs show concentration of the proteins of interest. Data are means ± SEM. The same results are represented as a heat map in figure 4a. $n = 4$.

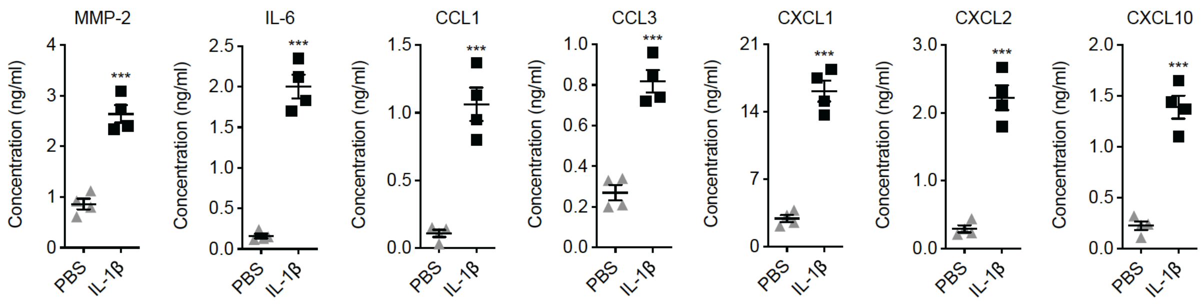

Supplementary Figure 9. Effect of IL-1β on senescence-associated secretory phenotype. Dermal fibroblasts were stimulated with IL-1β (1 ng/ml) for 24 h. The graphs show concentrations of senescence-associated secreted factors measured in the media. $n = 4$. Data are means ± SEM. Two-tailed Student's t test. $^{***}P \leq 0.001$.

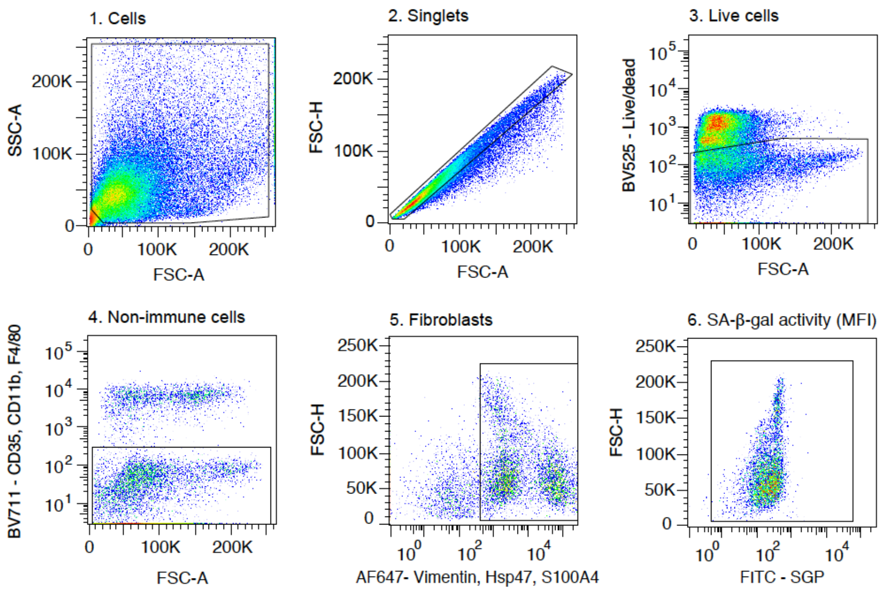

Supplementary Figure 10. Gating strategy to analyse β-gal activity in wound fibroblasts. Step by step representative flow cytometry dot plots are shown. MFI, median fluorescence intensity.

Recombinant proteins	Fibronectin K_d (nM)	Vitronectin K_d (nM)	Tenascin C K_d (nM)	Fibrinogen K_d (nM)
IL-1Ra	129.0 ± 7.2	73.2 ± 41.2	112.8 ± 13.0	662.7 ± 370.8
IL-1Ra/$PlGF_{123\text{-}141}$	21.9 ± 13.1***	0.7 ± 0.3*	20.3 ± 7.6***	43.5 ± 22.3*
PDGF-BB	67.5 ± 10.3	32.1 ± 3.6	94.3 ± 20.6	206.2 ± 85.7
PDGF-BB/$PlGF_{123\text{-}141}$	17.7 ± 2.0**	1.4 ± 0.1***	25.0 ± 2.9**	16.7 ± 2.0*

Supplementary Table 1. Binding-affinity of $PlGF_{123\text{-}141}$-fused IL-1Ra and PDGF-BB for ECM proteins. Dissociation constants (K_d) in nM are shown. K_d values of $PlGF_{123\text{-}141}$-fused proteins are all significantly lower compared to wild-type proteins. Data are means ± SD. n = 3 independent experiments. Two-tailed Student's t test. *$P \leq 0.05$, **$P \leq 0.01$, ***$P \leq 0.001$.
