# Supplementary material for: Restoration of the healing microenvironment in diabetic wounds with matrix-binding IL-1 receptor antagonist
Source: Commun Biol. 2021 Mar 26;4:422. doi: 10.1038/s42003-021-01913-9 (PMC7998035; doi:10.1038/s42003-021-01913-9)
Supplement: Supplementary file 3 — Description of Additional Supplementary Files [file 42003_2021_1913_MOESM3_ESM.pdf]

Description of Additional Supplementary Files

File Name: Supplementary Data 1

Description: Source data used to generate the graphs displayed in Figures 1 to 4 and Supplementary Figures 1, 3, 4, 5, 6, 8, and 9.
