# Supplementary material for: Restoration of the healing microenvironment in diabetic wounds with matrix-binding IL-1 receptor antagonist
Source: Commun Biol. 2021 Mar 26;4:422. doi: 10.1038/s42003-021-01913-9 (PMC7998035; doi:10.1038/s42003-021-01913-9)
Supplement: Supplementary file 5 — Reporting Summary [file 42003_2021_1913_MOESM5_ESM.pdf]

nature research

Corresponding author(s): Mikael M. Martino

Last updated by author(s): 21/02/21

Reporting Summary

Nature Research wishes to improve the reproducibility of the work that we publish. This form provides structure for consistency and transparency in reporting. For further information on Nature Research policies, see our Editorial Policies and the Editorial Policy Checklist.

Statistics

For all statistical analyses, confirm that the following items are present in the figure legend, table legend, main text, or Methods section.

n/a	Confirmed	
☐	☒	The exact sample size (*n*) for each experimental group/condition, given as a discrete number and unit of measurement
☐	☒	A statement on whether measurements were taken from distinct samples or whether the same sample was measured repeatedly
☐	☒	The statistical test(s) used AND whether they are one- or two-sided<br>*Only common tests should be described solely by name; describe more complex techniques in the Methods section.*
☐	☒	A description of all covariates tested
☐	☒	A description of any assumptions or corrections, such as tests of normality and adjustment for multiple comparisons
☐	☒	A full description of the statistical parameters including central tendency (e.g. means) or other basic estimates (e.g. regression coefficient) AND variation (e.g. standard deviation) or associated estimates of uncertainty (e.g. confidence intervals)
☐	☒	For null hypothesis testing, the test statistic (e.g. F, t, r) with confidence intervals, effect sizes, degrees of freedom and P value noted<br>*Give P values as exact values whenever suitable.*
☒	☐	For Bayesian analysis, information on the choice of priors and Markov chain Monte Carlo settings
☒	☐	For hierarchical and complex designs, identification of the appropriate level for tests and full reporting of outcomes
☒	☐	Estimates of effect sizes (e.g. Cohen's *d*, Pearson's *r*), indicating how they were calculated

Our web collection on statistics for biologists contains articles on many of the points above.

Software and code

Policy information about availability of computer code

Data collection	N/A
Data analysis	Prism Graphpad 7.0b, ImageJ1.51h, FlowJo 10.4.2, Zeiss ZEN 2.1, Microsoft Excel for Mac v16.16.8.

For manuscripts utilizing custom algorithms or software that are central to the research but not yet described in published literature, software must be made available to editors and reviewers. We strongly encourage code deposition in a community repository (e.g. GitHub). See the Nature Research guidelines for submitting code & software for further information.

Data

Policy information about availability of data

All manuscripts must include a data availability statement. This statement should provide the following information, where applicable:

- Accession codes, unique identifiers, or web links for publicly available datasets
- A list of figures that have associated raw data
- A description of any restrictions on data availability

The authors declare that all data supporting the results in this study are available within the paper and its Supplementary Information. The datasets generated and analysed during the study are available from the corresponding author upon reasonable request.

April 2020

Field-specific reporting

Please select the one below that is the best fit for your research. If you are not sure, read the appropriate sections before making your selection.

[x] Life sciences [ ] Behavioural & social sciences [ ] Ecological, evolutionary & environmental sciences

For a reference copy of the document with all sections, see nature.com/documents/nr-reporting-summary-flat.pdf

Life sciences study design

All studies must disclose on these points even when the disclosure is negative.

Sample size	The sample size (n) of each experiment is provided in the corresponding figure captions in the main manuscript and supplementary information files. Sample sizes were chosen to support meaningful conclusions. The effect size and standard deviation of the outcome were taken from similar experiments performed in the labs of the authors.
Data exclusions	No data was excluded from the analyses.
Replication	All in vitro experiments were replicated successfully at least 3 times.
Randomization	Mice were randomly distributed for each of the treatment types.
Blinding	For in vivo experiments, investigators were blinded to group allocation during data collection and analysis.

Reporting for specific materials, systems and methods

We require information from authors about some types of materials, experimental systems and methods used in many studies. Here, indicate whether each material, system or method listed is relevant to your study. If you are not sure if a list item applies to your research, read the appropriate section before selecting a response.

Materials & experimental systems

n/a	Involved in the study	
[ ]	[x]	Antibodies
[x]	[ ]	Eukaryotic cell lines
[x]	[ ]	Palaeontology and archaeology
[ ]	[x]	Animals and other organisms
[x]	[ ]	Human research participants
[x]	[ ]	Clinical data
[x]	[ ]	Dual use research of concern

Methods

n/a	Involved in the study	
[x]	[ ]	ChIP-seq
[ ]	[x]	Flow cytometry
[x]	[ ]	MRI-based neuroimaging

Antibodies

Antibodies used

1. Anti-F4/80 BV711, clone BM8, Biolegend 123147, 2 µg ml-1
2. Anti-CD11b Biotin, clone M1/70 ,Biolegend 101203, 2 µg ml-1
3. Anti-CD206 PE-Cy7, clone C0862C, Biolegend 141720, 2 µg ml-1
4. Anti-Ly6G BV421, clone 1A8, Biolegend 127628, 2 µg ml-1
5. Rat anti-CD11b BV711, clone M1/70, Biolegend 101241, 2 µg ml-1
6. Anti-CD45 BV711, clone 30-F11, Biolegend 103147, 1 µg ml-1
7. Anti-Vimentin biotin, clone EPR3776, Abcam ab254015, 0.2 µg ml-1
8. Anti-Hsp47, clone EPR4217, Abcam ab254015, 25 µg ml-1
9. Anti-S100A4, clone EPR14639, Abcam ab254015, 7.5 µg ml-1
10. Anti-CD31 Biotin, Abcam ab 124432, 0.8 µgml-1
11. Anti-Desmin Biotin, Abcam ab 8470, 1 µg ml-1
12. Anti-rabbit IgG Biotin, Thermo Fisher B2770, 1 µg ml-1
13. Alexa Fluor 594 Streptavidin, Life Technologies, S11227, 2 µg ml-1
14. Goat anti-mouse IgG Alexa Fluor 488, Abcam ab150117, 2 µg ml-1
15. Goat anti-rabbit IgG Alexa Fluor 647, Abcam ab 150083, 2 µg ml-1

Validation

For antibodies 1-9: serial dilutions were performed on spleen and skin tissues to determine optimal antibody dilutions for flow cytometry. For antibodies 10-15: dilutions were based on manufacturer recommendations for immunostaining.

Animals and other organisms

Policy information about studies involving animals; ARRIVE guidelines recommended for reporting animal research

Laboratory animals	C57BLKS/J-m/Lepr db mice (Jackson Laboratories) were males between 14-16 week old.<br>C57BL/6 mice (Jackson Laboratories) were males between 10-12 week old.
Wild animals	The study did not involve wild animals.
Field-collected samples	**N/A**
Ethics oversight	Monash University Animal Ethics Committee, Australia; Animal Research Committee of the Research Institute for Microbial Diseases of Osaka University, Japan.

Note that full information on the approval of the study protocol must also be provided in the manuscript.

Flow Cytometry

Plots

Confirm that:

- [x] The axis labels state the marker and fluorochrome used (e.g. CD4-FITC).
- [x] The axis scales are clearly visible. Include numbers along axes only for bottom left plot of group (a 'group' is an analysis of identical markers).
- [x] All plots are contour plots with outliers or pseudocolor plots.
- [x] A numerical value for number of cells or percentage (with statistics) is provided.

Methodology

Sample preparation	An 8 mm punch around the initial skin wound was isolated and enzymatically digested with 1mg ml-1 for immune profile and 1.5mg ml-1 for fibroblast senescence of Collagenase XI, 10ug ml-1 of Dnase I in complete RPMI (5% FBS, 2mM Glutamax). Collagenase was inactivated with PBS containing 5% FBS supplemented with 5mM EDTA. Tissue was passed through a 70µm filter and centrifuged at 300g for 10 mins. Cells were resuspended in Zombie aqua (Biolegend) in PBS for 30 mins on ice, washed and consecutively stained with TruStain FcX (Biolegend, 10µg ml-1 in PBS, 5% FBS) for 15 minutes at 4°C. Then cells were washed and stained with antibodies as described in the materials and methods.
Instrument	LSR Fortessa X20 Flow Cytometer (BD Biosciences)
Software	FlowJo Software V10.7.1 (Tree Star Inc)
Cell population abundance	At acquisition the entire 100% population is the population of interest.
Gating strategy	The gating strategy is provided in supplementary data.

- [x] Tick this box to confirm that a figure exemplifying the gating strategy is provided in the Supplementary Information.
